# Comparative Genomics of the *Listeria monocytogenes* ST204 Subgroup

**DOI:** 10.3389/fmicb.2016.02057

**Published:** 2016-12-22

**Authors:** Edward M. Fox, Theodore Allnutt, Mark I. Bradbury, Séamus Fanning, P. Scott Chandry

**Affiliations:** ^1^CSIRO Food and NutritionWerribee, VIC, Australia; ^2^CSIRO Food and NutritionNorth Ryde, NSW, Australia; ^3^UCD-Centre for Food Safety, School of Public Health, Physiotherapy and Sports Science, University College DublinDublin, Ireland

**Keywords:** *Listeria monocytogenes*, comparative genomics, MLST, whole genome sequencing, ST204, plasmid

## Abstract

The ST204 subgroup of *Listeria monocytogenes* is among the most frequently isolated in Australia from a range of environmental niches. In this study we provide a comparative genomics analysis of food and food environment isolates from geographically diverse sources. Analysis of the ST204 genomes showed a highly conserved core genome with the majority of variation seen in mobile genetic elements such as plasmids, transposons and phage insertions. Most strains (13/15) harbored plasmids, which although varying in size contained highly conserved sequences. Interestingly 4 isolates contained a conserved plasmid of 91,396 bp. The strains examined were isolated over a period of 12 years and from different geographic locations suggesting plasmids are an important component of the genetic repertoire of this subgroup and may provide a range of stress tolerance mechanisms. In addition to this 4 phage insertion sites and 2 transposons were identified among isolates, including a novel transposon. These genetic elements were highly conserved across isolates that harbored them, and also contained a range of genetic markers linked to stress tolerance and virulence. The maintenance of conserved mobile genetic elements in the ST204 population suggests these elements may contribute to the diverse range of niches colonized by ST204 isolates. Environmental stress selection may contribute to maintaining these genetic features, which in turn may be co-selecting for virulence markers relevant to clinical infection with ST204 isolates.

## Introduction

*Listeria monocytogenes* is a foodborne bacterial pathogen which causes listeriosis in humans (Allerberger and Wagner, 2010). This illness can manifest as a milder gastroenteritis form or a severe invasive infection which may include disease outcomes such as meningoencephalitis, sepsis, and stillbirth (Allerberger and Wagner, 2010). The bacterium has been isolated from a range of sources including the environment and foods, suggesting members of the species are capable of adaptation to diverse ecological niches (Gray et al., 2004; Fox et al., 2009; Freitag et al., 2009; Sauders et al., 2012).

With the increasing application of more sophisticated genomic analyses, a greater insight into *L. monocytogenes* population structure has been elucidated (Fox et al., 2012; Haase et al., 2014). The application of Multi Locus Sequence Typing (MLST) has provided insights into the evolution of the species and identified associations of subgroups to certain environmental niches or clinical illness (Unterholzner et al., 2013; Haase et al., 2014; Maury et al., 2016). Dominant sequence types (STs) and clonal complexes (CCs; groups of closely related STs) have been identified both in a geographical context and as well as with source associations (e.g., clinical or food sources; Chenal-Francisque et al., 2011; Haase et al., 2014; Ebner et al., 2015; Maury et al., 2016). These studies have, for example, identified CC1/ST1 as a dominant subgroup at both a national or global level with strong clinical significance. Similar analysis of *L. monocytogenes* isolates from France identified CC121 as the most common ST, which was predominantly associated with food (Maury et al., 2016). Although analysis of *L. monocytogenes* isolates from Australia has identified dominant subgroups also over-represented in other geographical regions, one notable exception is the ST204 subgroup (Kwong et al., 2016). This subgroup is among the most common ST identified in Australia, is associated with a diverse range of food production environments and products and also with human clinical infection. In contrast to this, it has been reported at lower frequency relative to other ST subgroups in studies outside Australia and with the exception of data from France, usually only in association with food sources (Haase et al., 2014; Ebner et al., 2015).

Whole genome sequencing has facilitated new insights into the genomics of *L. monocytogenes* and has allowed greater understanding of traits shared across the species along with those characteristic of subgroups of *L. monocytogenes* (den Bakker et al., 2010; Schmitz-Esser et al., 2015; Maury et al., 2016). Studies such as these have facilitated a greater understanding of aspects such as strain virulence and stress resistance mechanisms (Nightingale et al., 2005; Muller et al., 2013). It is now becoming apparent, for example, that certain subgroups of *L. monocytogenes* may be less pathogenic than others, or may contain mechanisms that promote other phenotypes such as persistence in localized environments (Cotter et al., 2008; Van Stelten et al., 2010; Verghese et al., 2011). Genetic variation between closely related isolates can yield information on traits characteristic of such subgroups; analysis of the ST121 subgroup, for example, identified conserved genetic features including a transposon mediating resistance to quaternary ammonium compound-based sanitizers, as well as highly conserved phage and plasmids (Schmitz-Esser et al., 2015).

This study provides a genomic analysis of the *L. monocytogenes* ST204 subgroup, a prevalent subgroup which has colonized a range of environmental niches in Australia and is linked with sporadic illness in humans. A panel of 15 isolates were chosen to represent diverse sources (foods including milk, cheese, and pork, as well as food production environments), different geographical locations (including the Australian States of New South Wales, South Australia, Tasmania, Victoria and Western Australia, as well as an isolate from Ireland) and different isolation dates (ranging from 2000 to 2015). This study includes characterization of mobile genetic elements harbored by ST204 isolates from Australia and abroad, including phage, transposons and plasmids.

## Materials and methods

### Isolates included in this study

Table 1 lists the 15 isolates included in this study. To understand the high prevalence of this subgroup noted in food and food environment niches, isolates were selected from a range of food or food processing facility environments (including dairy and meat foods, as well as food production environments). Fourteen isolates originated in Australia and one was isolated from a meat production facility in Ireland. Australian isolates were sourced from five different States: New South Wales (*n* = 5), South Australia (*n* = 1), Tasmania (*n* = 1), Victoria (*n* = 6), and Western Australia (*n* = 1). The years of isolation ranged from 2000 to 2015.

**Table 1:** **Summary of the isolates in this study and overview their associated genetic features**.

	**Isolation**				**Genome**					**Transposons**		**Phage inserts**			
**Isolate**	**Source**	**Country**	**State^a^**	**Year**	**Size (bp)**	**GC Content (%)**	**Number of CDS**	**CDS % of genome**	**Plasmid^b^**	**Tn*yfbR***	**Tn*ILP***	**φ*comK***	**φMonocin**	**φtRNA-Arg**	**φRNA-MT**
2882	Meat-Pork	Australia	VIC	2000	3,037,029	37.8	3001	90.2	91,396 bp	–	+	+	+	−	−
2919	Meat-Environment	Australia	VIC	2007	3,113,342	37.7	3096	90.3	+	+	+	+	+	−	−
2937	Dairy-Cheese	Australia	NSW	2009	2,949,465	37.8	2886	90.4	47,340 bp	–	+	−	+	−	−
2939	Dairy-Cheese	Australia	NSW	2009	2,948,045	37.8	2910	90.4	+	–	+	−	+	−	−
2945	Dairy-Cheese	Australia	TAS	2010	3,084,502	37.9	3063	90	91,396 bp	+	+	+	+	−	−
2964	Dairy-Cheese	Australia	VIC	2011	3,044,927	37.8	3010	90.2	+	+	+	+	+	−	−
2973	Dairy-Cheese	Australia	VIC	2012	3,049,747	38.0	3064	89.6	38,115 bp	–	−	+	+	+	+
2977	Dairy-Cheese	Australia	VIC	2012	2,990,589	37.8	2920	89.9	+	+	+	−	+	−	−
2978	Dairy-Cheese	Australia	VIC	2012	3,029,511	37.8	2987	90	+	+	+	+	+	−	−
2981	Dairy-Milk	Australia	SA	2012	3,081,990	37.8	3060	90	91,396 bp	−	+	+	+	+	−
3002	Dairy-Environment	Australia	WA	2006	2,983,094	37.8	2931	90.2	–	+	+	+	+	−	−
Lm15-001	Meat-Environment	Australia	NSW	2015	2,972,510	37.9	2979	90.6	38,191 bp	−	−	+	+	+	−
Lm15-011	Meat	Australia	NSW	2015	3,013,664	37.8	2936	89.8	+	+	+	−	+	−	−
Lm15-027	Meat	Australia	NSW	2015	2,953,353	38.0	2939	90.5	–	−	−	−	+	+	+
UCDL175	Meat-Environment	Ireland	n/a	2012	3,020,537	37.8	2966	90.5	91,396 bp	+	+	−	+	−	−

a *VIC, Victoria; NSW, New South Wales; TAS, Tasmania; SA, South Australia; WA, Western Australia; n/a, not applicable*.

b *Plasmids whose genome has not been closed are represented with a “+” symbol*.

### Whole genome sequencing, assemblies and annotations

Genomic DNA was extracted from isolates using the DNeasy Blood and Tissue kit (QIAGEN, Hilden, Germany) according to the manufacturer's instructions. DNA quantity was measured using the Qubit dsDNA HS assay kit (Thermo Scientific, Waltham, MA), and an A260/A280 of 1.8–2.0 was confirmed using a NanoDrop spectrophotometer (Thermo Scientific, Waltham, MA). DNA preparations were sent to the Ramaciotti Centre for Genomics (University of New South Wales, Sydney, Australia) for sequence ready genomic library preparation using the Nextera XT library prep kit (illumina, San Diego, CA). Subsequently 300 bp paired-end sequencing was performed using the illumina MiSeq platform. Raw reads were pre-processed to remove adapter sequences and low quality reads using the Trimmomatic version 0.22 software (Bolger et al., 2014). De novo assembly was performed using the SPAdes (Species Prediction and Diversity Estimation) genome assembler tool version 2.5.1 based on an algorithm which employs multisized De bruijn graphs with K-mer values of “21, 33, 55, and 77” to construct the contiguous sequences (contigs) (Bankevich et al., 2012). FASTA files generated were processed through the online gene annotator RAST (Rapid Annotation of microbial genomes using Subsystems Technology) to produce GENBANK files (Aziz et al., 2008). Genome analysis including mobile element alignments was performed using Geneious software (Kearse et al., 2012). This Whole Genome Shotgun project has been deposited at DDBJ/ENA/GenBank under the accessions: LXQP00000000 (strain 2882); LXQQ00000000 (strain 2919); LXQR00000000 (strain 2937); LXQS00000000 (strain 2939); LXQT00000000 (strain 2945); LXQU00000000 (strain 2964); LXQV00000000 (strain 2973); LXQW00000000 (strain 2977); LXQX00000000 (strain 2978); LXQY00000000 (strain 2981); LXQZ00000000 (strain 3002); LXRA00000000 (strain Lm15-001); LXRB00000000 (strain Lm15-011); LXRC00000000 (strain Lm15-027); LXRD00000000 (strain UCDL175). The versions described in this paper are versions LXQP01000000, LXQQ01000000, LXQR01000000, LXQS01000000, LXQT01000000, LXQU01000000, LXQV01000000, LXQW01000000, LXQX01000000, LXQY01000000, LXQZ01000000, LXRA01000000, LXRB01000000, LXRC01000000, LXRD01000000, respectively.

### Comparative genomic analysis

To visualize comparative BLAST alignments, chromosomes were compared using an in-house BLAST ring alignment python script, circles1.2.py (https://github.com/tallnuttcsiro/circles); plasmids were compared using BRIG software (Alikhan et al., 2011); transposon and phage alignments were visualized using Easyfig software (Sullivan et al., 2011). SNP analysis was performed using the Parsnp program, part of the Harvest suite (Treangen et al., 2014). To construct an ordered chromosome pangenome, an optical map was generated for isolate UCDL175 using the Argus optical mapping system and MapSolver software (OpGen, Gaithersburg, MD). Assembled contigs were ordered by mapping to an *Nco*I genome restriction map. To construct a plasmid pangenome, unique features across all plasmids were combined into a single contig, ordered based on the largest closed plasmid pUCDL175.

## Results and discussion

*Listeria monocytogenes* can occupy a diverse set of environmental niches in addition to sporadic carriage in animal and human hosts where it may also cause associated infection (Vazquez-Boland et al., 2001; Grif et al., 2003; Ho et al., 2007; Sauders et al., 2012). Geographical or niche-specific variations have been identified in relation to associations with certain subgroups, for example ST9 and ST121 are highly represented among food isolates in many jurisdictions (Chenal-Francisque et al., 2011; Ebner et al., 2015). An interesting observation in the distribution of *L. monocytogenes* STs among isolates from Australia is the high relative proportion of ST204 isolates, which have not been identified at this high relative abundance in other geographical populations (Chenal-Francisque et al., 2011; Haase et al., 2014; Kwong et al., 2016). In addition, a diverse range of clinical and non-clinical sources are associated with these ST204 isolates, including various foods and the environment. This study interrogated draft genome sequences of representative isolates from this dominant subgroup of *L. monocytogenes* identified in Australia to understand their associated genetic characteristics and identify the presence of genetic markers related to clinical illness and/or environmental stress.

### Overview of the ST204 pangenome

**Figure 1:**
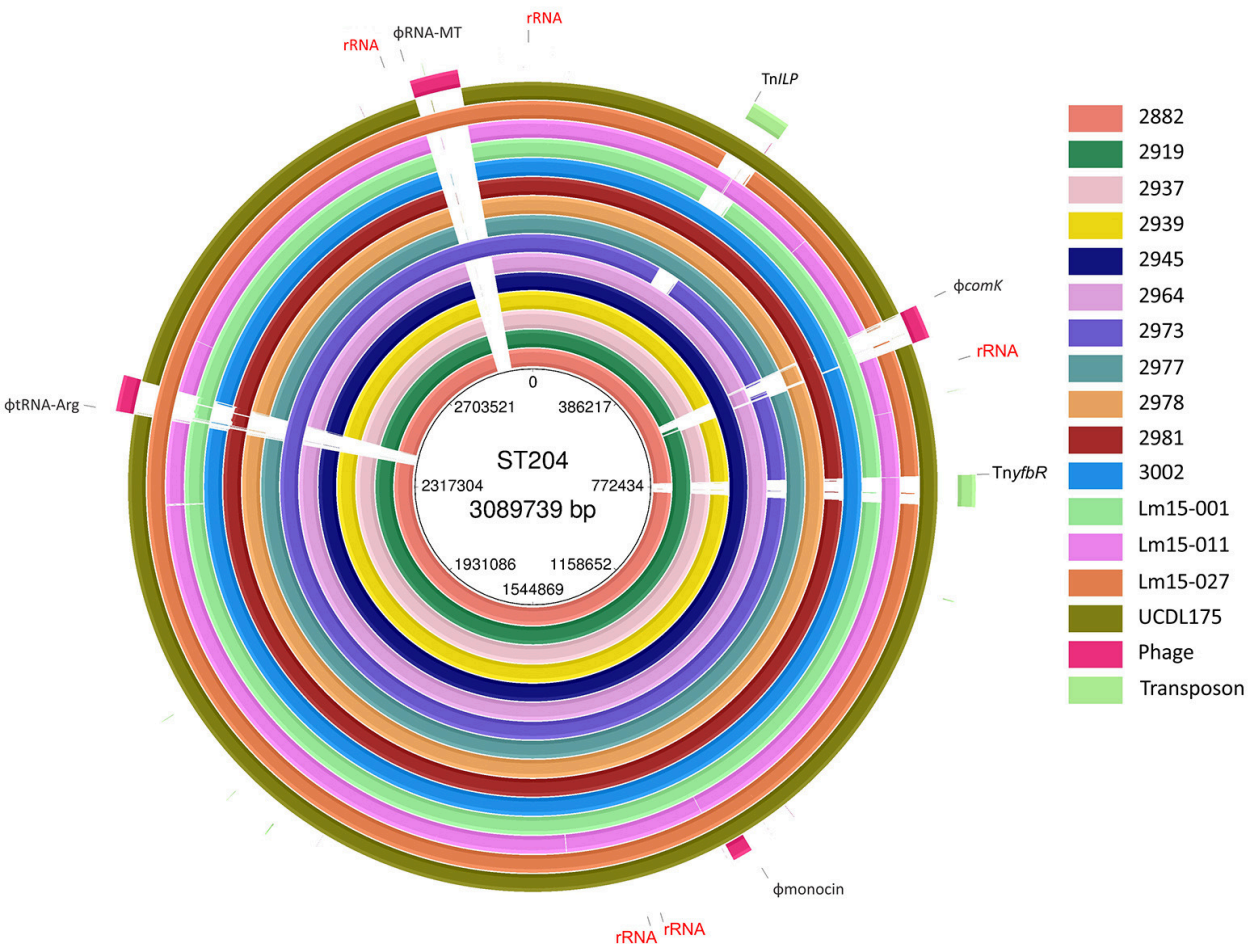
**BLAST ring representation of the chromosome features of isolates in this study**. Each ring represents the chromosome of a single isolate, BLAST aligned to a pangenome reference which includes all genetic features of isolates combined into a single ordered contig reference. Ribosomal RNA regions are labeled “rRNA.” Transposons: Tn*yfbR* and Tn*ILP*. Phage locations are identified by the “Phi” prefix.

**Figure 2:**
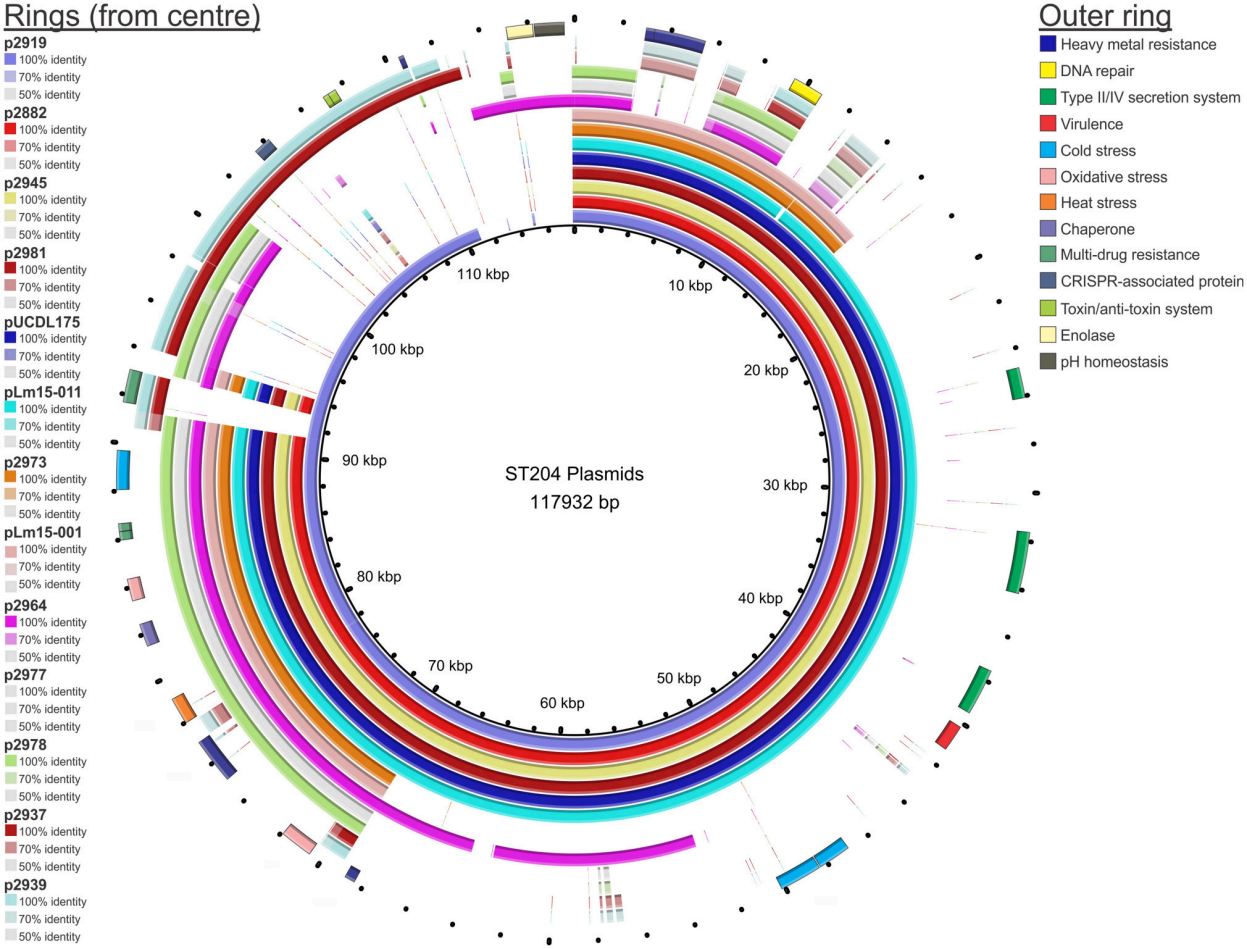
**BRIG representation of the plasmids of isolates in this study**. Each ring represents the plasmid of an individual isolate. The plasmid pangenome reference contains all unique genetic markers identified among the entire pool of plasmids identified, combined into a single contig reference. The % identity refers to the similarity to the plasmid reference pan-genome.

A summary of the genomes of the 15 ST204 isolates included in this study is shown in Table 1. Genome sizes ranged from 2.95 to 3.11 Mbp, with a GC content ranging from 37.7% up to 38%. The number of gene CDSs (coding DNA sequence) ranged from 2886 in the smallest genome to 3096 in the largest genome. A core genetic backbone was shared by all ST204 isolates in this study; where greatest variation was observed in the presence or absence of mobile genetic elements such as plasmids, transposons or phage insertions (Figures 1, 2). Plasmids were present in 13 of the 15 isolates. There were four phage insert regions identified among isolates, and two transposon insertions (Figure 1).

### Classical *L. monocytogenes* virulence and stress tolerance markers

The LIPI-1 pathogenicity island (Vázquez-Boland et al., 2001), comprising key virulence genes, contained a single SNP when compared to that of the EGD-e Type strain (Figure S1). This SNP was positioned in the metalloprotease gene *mpl*, which plays a role in intracellular survival; the SNP resulted in a conservative amino acid (AA) change (D → E) at position 460 in the gene, outside the active site and zinc binding domains. Previous studies have identified two notable mutations in important *L. monocytogenes* virulence markers: truncation of the InlA invasion protein and a deletion in the ActA protein which plays a role in intracellular mobility and cell to cell spread (Jonquières et al., 1998; Moriishi et al., 1998; Nightingale et al., 2005). Analysis of the ST204 genomes of isolates in this study showed that they all encode for full length InlA and ActA proteins. None of the ST204 isolates harbored the LIPI-3 or the newly described LIPI-4 pathogenicity islands (Cotter et al., 2008; Maury et al., 2016).

All ST204 isolates contained the *L. monocytogenes* stress survival islet SSI-1 and shared 100% nucleotide identity to the EGD-e strain, with one exception (Ryan et al., 2010). The ST204 2981 isolate contained a single nucleotide deletion in its *lmo0446* homolog leading to the occurrence of a premature stop codon (PMSC) in the gene sequence, which suggests it produces a truncated 172 AA protein (the wild type protein being 330 AA in length). Nucleotide sequence analysis suggests the 172 AA product to still include the complete catalytic domain and contains all the conserved amino acids of a penicillin V acylase. The *bcrABC* resistance cassette, which contributes to resistance to quaternary ammonium compounds (QACs) was present on the plasmids of 11 isolates as indicated in Table 2 (Elhanafi et al., 2010). The sequence shared 100% identity to that of the corresponding region on the pN1-011A plasmid of the N1-011A strain. These genetic markers provide resistance to a range of stresses and growth limiting conditions including sanitizers used in food processing environments (Elhanafi et al., 2010; Ryan et al., 2010). Taken together, these resistance markers point to the capacity of the ST204 group to colonize a wide range of niches with various associated stress conditions.

**Table 2:** **Presence of selected virulence and stress response markers among isolates in this study**.

	**Virulence**				**Stress tolerance**		
**Isolate**	**LIPI-3**	**LIPI-4**	***inlA***	***actA***	**SSI-1**	***bcrABC***	***qacH***
2882	–	–	WT^a^	WT	+	+	−
2919	–	–	WT	WT	+	+	−
2937	–	–	WT	WT	+	−	−
2939	–	–	WT	WT	+	−	−
2945	–	–	WT	WT	+	+	−
2964	–	–	WT	WT	+	+	−
2973	–	–	WT	WT	+	+	−
2977	–	–	WT	WT	+	+	−
2978	–	–	WT	WT	+	+	−
2981	–	–	WT	WT	+^b^	+	−
3002	–	–	WT	WT	+	−	−
Lm15-001	–	–	WT	WT	+	+	−
Lm15-011	–	–	WT	WT	+	+	−
Lm15-027	–	–	WT	WT	+	−	−
UCDL175	–	–	WT	WT	+	+	−

*SSI-1 is the stress-survival islet. The bcrABC cassette and qacH are quaternary ammonium compound resistance markers*.

a *WT, codes full-length protein with no truncation or deletion*.

b *Isolate 2981 contains a PMSC in its lmo0446 homolog*.

### ST204 plasmids

A comparative BLAST analysis of plasmids from each of the 13 plasmid-containing isolates against a plasmid pangenome pseudomolecule is shown in Figure 2. With the exception of isolates 2937 and 2939, plasmids from other isolates shared an approximately 20 kb region which contained a number of genes linked to stress response. Analysis of the plasmids identified a 91,396 bp plasmid shared by four isolates: p2882, p2945, p2981, and pUCDL175. The plasmid nucleotide sequence was identical for p2882, p2945, and p2981, with one single nucleotide polymorphism (SNP) difference in pUCDL175. This SNP resulted in a non-conservative AA change in a DEAD/DEAH box helicase-like protein at position AA_332_ (pUCDL175 had an isoleucine at this position compared to a threonine in the other isolates). The presence of a conserved plasmid among the four isolates was particularly interesting as one of these isolates, UCL175, was derived from a meat food processing environment in Ireland; the other three were isolated in Australia from a pork meat sample, a meat food processing environment, and a cheese sample. In addition to this, the temporal range of the isolations was over a 12 year period (from the year 2000 through to 2012). This data suggests selection pressure has maintained this plasmid in geographically diverse niches and over a prolonged period of time; with just a single SNP identified in the plasmid of the isolate from Ireland, relative to its three counterparts from Australia. The plasmid contains features of a Type IV secretion system including Tra-family proteins which play a role in conjugal transfer of plasmids in Gram-positive bacteria (Grohmann et al., 2003). Another feature of this and other plasmids identified in this study is the presence of heavy metal resistance gene operons. Recent evidence suggests very low levels of heavy metals and antibiotics present in the environment can select for plasmid carriage in bacterial species (Gullberg et al., 2014). It may be that this, or other selection factors, while even at very low levels are maintaining the presence of these plasmids in the ST204 isolates and their progeny. As indicated in Figure 2, a number of other genes implicated in stress response were identified such as cold, heat, or osmotic stress. Maintaining these plasmids may present a competitive advantage for these isolates, allowing them to colonize and compete in a diverse range of ecological niches and tolerate a range of environmental stressors.

A number of other plasmids shared homology with the 91,396 bp plasmid (the smaller plasmids p2973 and pLm15-011, and the larger p2919 plasmid). This may indicate a shared ancestral plasmid that has undergone genetic rearrangement and divergence over time. Indeed, the remaining plasmids (p2964, p2977, p2978, p2937, and p2939) shared most of their sequence with p2919, with some minor additional regions as indicated in Figure 2. The pLm15-011 plasmid from isolate Lm15-011 had an identical sequence to that of p2882, p2945, and p2981, with the exception of a missing 213 bp (this deleted region included a non-coding region as well as the first 29 bp of a plasmid replication protein). Another variant of the 91,396 bp plasmid was identified in isolate 2919, which contained a larger plasmid with additional genetic features including a CRISPR-associated protein and a toxin/anti-toxin (TA) system (p2919, Figure 2). This was identified as the Type II Phd-Doc TA system, whose mode of action targets the 30S ribosomal subunit inhibiting translation elongation (Liu et al., 2008; Unterholzner et al., 2013). Two isolates harbored smaller homologs of the 91,396 bp plasmid: 2973 and Lm15-001. p2973 is 38,115 bp in length and shares 100% identity with nucleotide sequence contained within the 91,396 bp plasmid. pLm15-001 is 38,191 bp in size, and differs with p2973 in that it contains a 76 bp insert and four SNPs in a non-coding region of the plasmid; all CDSs, however, are 100% identical in both plasmids.

The remaining plasmids (p2964, p2977, p2978, p2937, and p2939) are primarily composed of various homologs of other sequence contained in p2919 as illustrated in Figure 2; p2964, however, does contain two notable additional CDSs encoding enolase and a gene implicated in pH homeostasis. Two of these plasmids (p2937 and p2939) also contained the Type II Phd-Doc TA system present in p2919. It is worth noting that these ST204 plasmids share a high degree of similarity to the large 148,959 bp pN1-011A plasmid, suggesting variations of this plasmid may be spread more widely across the *L. monocytogenes* population.

### Transposon inserts

**Figure 3:**
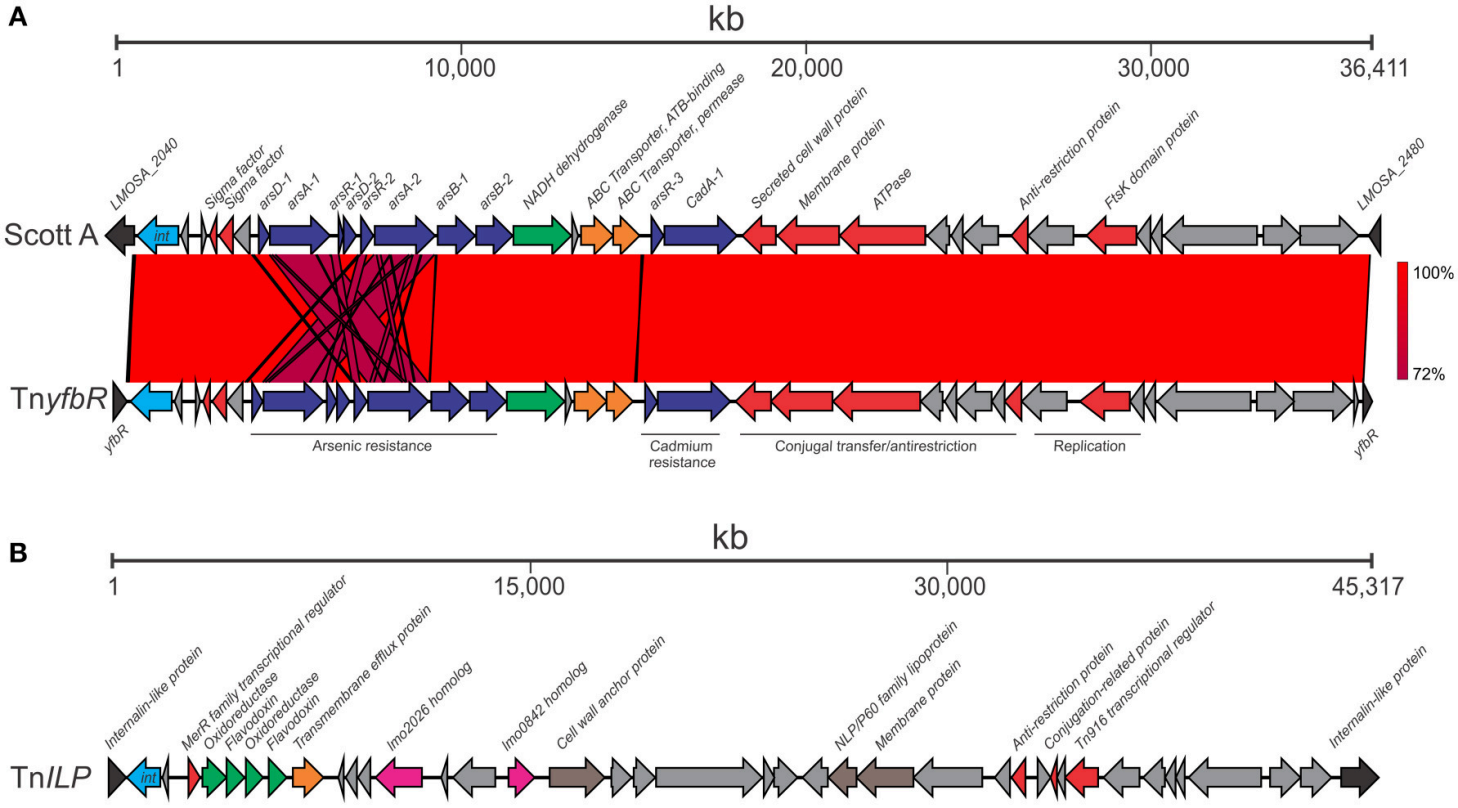
**Transposons identified among ST204 isolates**. Two transposons were identified: **(A)** The Tn*yfbR* transposon inserted in the *yfbR* gene compared with its Scott A strain homolog; **(B)** The Tn*ILP* transposon inserted in an internalin-like protein. The integrase genes are in light blue (marked “*int*”); other transposon system and regulatory genes are in red; heavy metal resistance genes are in dark blue; oxidative stress are in green; membrane associated proteins are brown; virulence proteins are in pink; ABC transporter system genes are in orange. Hypothetical gene or those without a known function are shaded gray. Blue lines represent similar BLAST identity shared between genes in the arsenic resistance cassette. Sequence identity ranged from 72 to 100% as indicated by the percentage homology bar.

Two transposon insertions were identified among isolates in this study: one in the *yfbR* gene (Tn*yfbR*) and another in an internalin-like protein homolog of the *lmo2026* gene in the EGD-e strain (Tn*ILP*). The organization of both transposons are shown in Figure 3.

The 35,763 bp Tn*yfbR* transposon was present in eight isolates (Table 1 and Figure 1). This transposon is homologous to a previously identified genomic island in the Scott A outbreak strain (Figure 3A; Lee et al., 2013). Four SNPs were identified between the Scott A and ST204 transposon, however the insert site differed between ST204 isolates that harbored the genomic island and Scott A. This may underly results from the study by Lee et al. (2013) suggesting multiple loci for insertion across different strains. It contains an arsenic resistance cassette, which includes the *arsA-1, arsA-2, arsB-1, arsB-2, arsD-1, arsD-2, arsR-1*, and *arsR-2* genes. *arsA* and *arsB* form a complex coupled with ATP which is responsible for extrusion of arsenic from the bacterial cell (Rosen, 2002). Expression is regulated by *arsR* and *arsD* (San Francisco et al., 1990; Wu and Rosen, 1993). This transposon also contains a cadmium resistance cassette. Other notable features include an anti-restriction protein and an FtsK domain protein.

A second novel transposon was identified in 12 ST204 isolates, termed Tn*ILP*, and was identified integrated into an internalin-like protein (Figure 3B). It is 43,478 bp in length and contains a number of proteins involved in electron transfer such as flavodoxin and oxidoreductase CDSs, including 2,5-diketo-D-gluconic acid reductase. These enzymes have a variety of associated functions, including the oxidative stress response (Gaudu and Weiss, 2000; Mbandi et al., 2007; Chaturongakul et al., 2008; Moyano et al., 2014). This transposon also contained a number of genes encoding cell wall associated proteins including internalin-like LPXGT-motif containing proteins. Two other notable gene homologs of the EGD-e strain, *lmo0842* and *lmo2026*, were present in Tn*ILP*. Previous studies using transposon mutagenesis approaches have identified roles for these genes in virulence. An *lmo0842* mutant strain showed decreased ability to proliferate in the liver and spleen (Cummins et al., 2013), whereas a role for *lmo2026* in translocating across the blood-brain barrier and/or subsequent multiplication has been suggested (Autret et al., 2001; Bierne et al., 2007). The potential contribution of the Tn*ILP* genes to stress response and virulence, however, requires further study.

### Phage insert regions

Four phage insert sites were identified among isolates in this study: φ*comK*, the monocin locus cryptic prophage, φtRNA-Arg and φRNA-MT. Analysis with prophage detection program (PHAST) suggests the only intact phage is φtRNA-Arg; all others were incomplete phage. All isolates contained at least one phage insert, with a single isolate (2973) containing phage in all four insertion sites. Of the four prophage insertions, two were highly conserved: the monocin locus phage region (present in all isolates), and a phage insert positioned between the fosfomycin resistance gene fosX and an RNA methyltransferase gene (φRNA-MT, present in both 2973 and Lm15-027 isolates).

Two isolates harbored the φRNA-MT phage in their RNA methyltransferase genes (2973 and Lm15-027) with a single SNP in a non-coding sequence being the only difference in nucleotide sequence. This phage is 56,942 bp in length and encodes a number of restriction-modification system genes, including both Type I and Type II methyltransferases. These systems can target foreign invading DNA with restriction endonucleases, and associated methyltransferases protect host DNA from restriction (Wilson and Murray, 1991; Murphy et al., 2013). A HNH family endonuclease is also present in this phage. There are a range of functions associated with these proteins such as excluding other phage in the progeny of cells with mixed phage infections (Goodrich-Blair and Shub, 1996; Moodley et al., 2012). The phage also contained an oxidoreductase.

**Figure 4:**
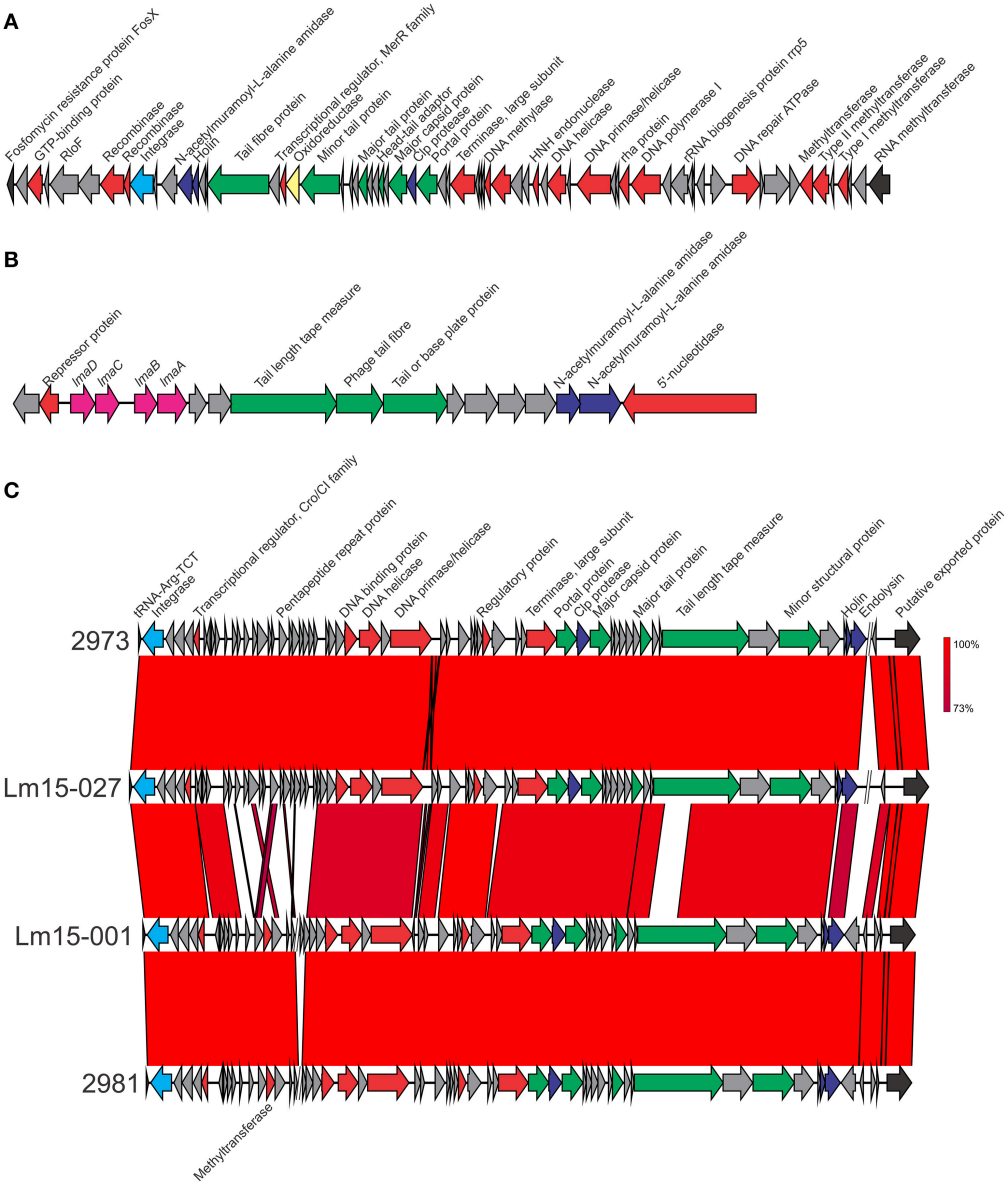
**Genetic makers of 3 phage inserts identified in isolates in this study. (A)** φRNA-MT phage upstream of an RNA methyltransferase gene. **(B)** The monocin phage locus. **(C)** phage insert downstream of tRNA-ARG. Integrase genes are colored light blue, phage structural genes in green, phage non-structural genes in red, proteases in dark blue, virulence genes in pink and oxidative stress genes in yellow. Where sequence identity was shared, this ranged from 73 to 100% as indicated by the percentage homology bar.

The monocin locus (Figure 4B) is a cryptic prophage conserved across all lineages of *L. monocytogenes* and harbors a complete (*lmaDCBA*) or partial *lma* operon (*lmaDC*) (Schäferkordt and Chakraborty, 1997; Hain et al., 2012). LmaA elicits a hypersensitivity reaction in the immune murine host model, and deletion mutants of *lmaB* and *lmaD* show reduced growth in the model (Hain et al., 2012). This phage is 13,114 bp in length and includes 18 CDSs. This region showed 100% nucleotide sequence identity across all isolates and shared 99% identity to the same region in the EGD-e strain.

The only intact phage, downstream of tRNA-Arg, was 42,164 bp in length with similar variants identified in four isolates: 2973, 2981, Lm15-001, and Lm15-027. The annotated phage region contained a large proportion of genes coding for proteins with unknown function (Figure 4C). While largely conserved across all four isolates, some variations were identified in the phage insert regions (e.g., 2981 and Lm15-001 contained a methyltransferase coding gene not present in the other two isolates).

**Figure 5:**
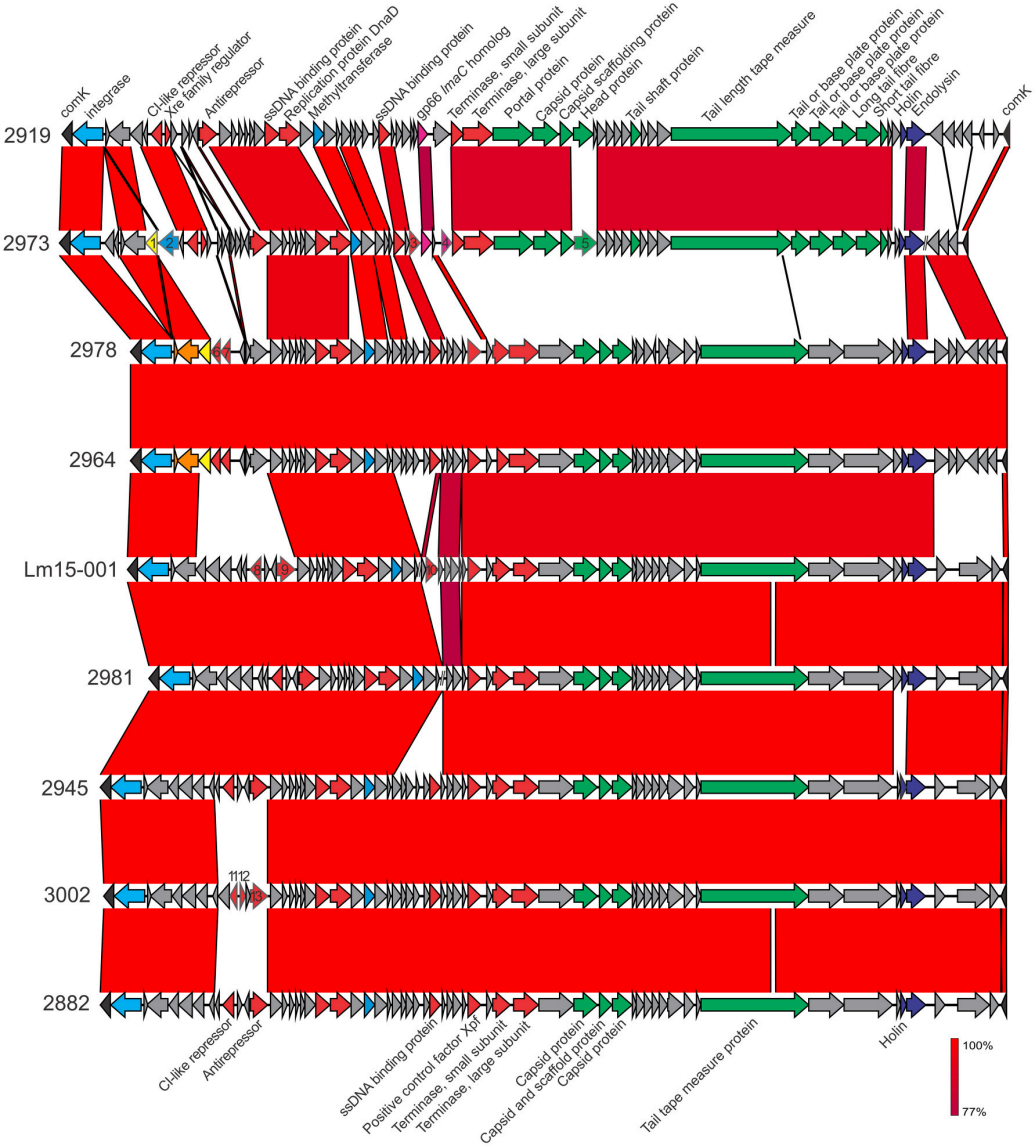
**Comparative analysis of *comK* phage inserts among ST204 isolates in this study**. Genes marked by number: 1, putative serine protease; 2, putative superinfection immunity protein; 3, RecU Holliday junction resolvase; 4, *lmaC*; 5, phage capsid protein; 6, phage repressor protein; 7, phage transcriptional regulator, Cro/CI family; 8, repressor (CI-like); 9, antirepressor; 10, HNH homing endonuclease; 11, repressor (CI-like); 12, transcriptional regulator; 13, phage antirepressor protein. Phage structural or non-structural genes are colored green or red, respectively. Integrases are colored light blue, virulence-associated genes in pink, phage proteases in dark blue. Where sequence identity was shared, this ranged from 77 to 100% as indicated by the percentage homology bar.

A phage insert (φ*comK*) in the *comK* gene (Figure 5) was identified in seven isolates, ranging in size from 37,660 bp (isolate 2981) up to 41,592 bp (isolate 2919). Isolates 2964 and 2978 differed from one another in a single SNP present in an *lmo2305* homolog “hypothetical protein” gene in an amino acid change at position 73 (E → G) in 2978; however each other isolate had a distinct *comK* phage region as illustrated in Figure 4. Two isolates (2919 and 2973) contained a gene encoding the gp66 protein identified in the *L. monocytogenes* A118 phage (Loessner et al., 2000). This protein shares similarity with the virulence protein LmaC of the monocin phage locus. Interestingly the 2973 isolate also contained another copy of the *lmaC* gene two genes downstream of its gp66 gene. A previous study highlighted the importance of this phage region in host strain escape from activated macrophages by modulating excision of the phage, which in turn controls *comK* integrity (Rabinovich et al., 2012). This excision, however, does not create virions. Another proposed role of the *comK* phage in host cell adaption to specific niches is in colonization of food processing facilities and foods (Verghese et al., 2011). While all isolates in this study were from foods or their associated processing environments, it is not clear if they were persisting at these facilities. The *comK* phage in isolate 2973 also coded a superinfection immunity protein which may play a role in preventing subsequent host infection by other phage (Hyman and Abedon, 2010). All *comK* phage also contained a methyltransferase gene. Isolate 2973 contained a superinfection immunity gene not present in the *comK* phage of other isolates. Three isolates (2964, 2973, and 2978) possess a serine protease encoding gene, and Lm15-001 *comK* encoded a HNH homing endonuclease.

### CRISPR regions among isolates in this study

An analysis of each of the 15 isolates in this study using CRISPRfinder software did not identify any confirmed CRISPR regions (Grissa et al., 2007). Two isolates harbored a CRISPR-associated protein on their respective plasmids (2937 and 2939) although the plasmids did not contain CRISPR regions (Figure 2).

### SNP typing of isolates in this study

**Figure 6:**
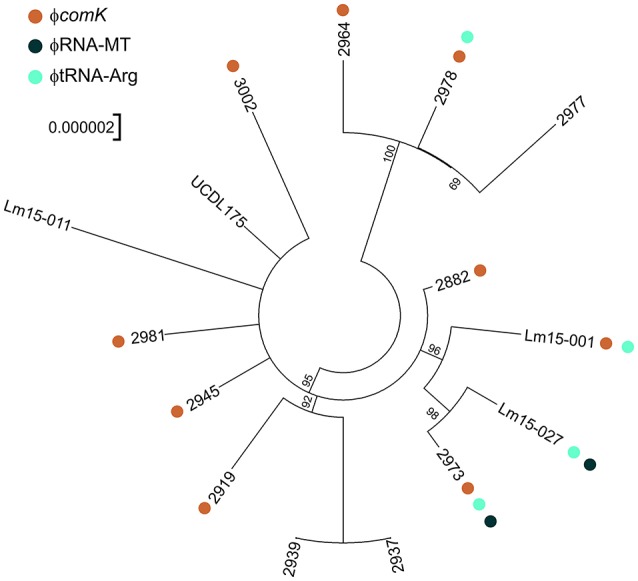
**SNP tree analysis of ST204 isolates in this study**. SNP phylogeny is unrooted but has been plotted as a circular phylogram to improve readability. Presence of prophage is marked by colored dots as per legend with the exception of ϕMonocin which is present in all isolates. Bootstrap values above 50 are displayed.

Analysis of the genome similarities through a SNP analysis again highlighted the high degree of identity among the isolates, with 266 SNP loci identified (Figure 6). While three clades can be readily discerned from the SNP analysis (clade 1 comprising isolates 2964, 2977, and 2978; clade 2 comprising 2973, Lm15-001 and Lm15-027; and clade 3 comprising 2919, 2937 and 2939) the remainder of the taxonomy had a high degree of uncertainty. The φRNA-MT phage was only identified among clade 2 isolates, and similarly the φtRNA-Arg phage was only identified among isolates from clades 1 and 2. Two isolates (2937 and 2939) clustered together when considering core SNP loci shared by all 15 isolates; a comparison of SNPs limited to these 2 isolates alone identified 24 SNPs differing between them.

## Conclusions

The ST204 subgroup of *L. monocytogenes* is among the most frequently isolated from food and food associated sources in Australia, has been isolated from other environments and has been implicated in human clinical infections. Results of this study show a diverse accessory genome conserved among ST204 isolates including genetically related plasmids, transposons and phage inserts which appear to be maintained among the geographically diverse population over prolonged periods of time. Recent studies have demonstrated how selective pressures, often below minimum inhibitory concentrations, can select for bacteria possessing associated resistance determinants (Gullberg et al., 2011). In addition to this, co-selection is often evident when multiple resistance genes (e.g., heavy metals and antibiotics) are harbored on the same genetic element, such as a plasmid (Gullberg et al., 2014). The broad range of antimicrobial and stress resistance genes harbored by ST204 isolates may underlie their capacity to colonize a wide range of niches. This adaptation to a broad range of environmental stress conditions may also be co-selecting for a range of associated virulence markers which has implications for the clinical significance of the ST204 group.

## Author contributions

EF, SF, and PSC conceived and designed the experiments. EF, MB performed all wet laboratory experiments. EF, TA, and PSC conducted bioinformatics analyses. EF, TA, MB, SF, and PSC drafted manuscript. All authors read and approved final manuscript, and agree to be accountable for all aspects of the work in ensuring that questions related to the accuracy or integrity of any part of the work are appropriately investigated and resolved.

## Funding

This work was co-funded by the Victorian Government through the Department of Environment and Primary Industries and by the Commonwealth Scientific and Industrial Research Organization.

### Conflict of interest statement

The authors declare that the research was conducted in the absence of any commercial or financial relationships that could be construed as a potential conflict of interest.

## Supplementary material

Figure S1 **Nucleotide alignment of the conserved serotype 1/2a ST204 LIPI-1 pathogenicity island with the serotype 1/2a ST35 EGD-e reference strain**.
