# Supplementary figures and images for: Comparative Genomics of the Listeria monocytogenes ST204 Subgroup
Source: Front Microbiol. 2016 Dec 22;7:2057. doi: 10.3389/fmicb.2016.02057 (PMC5177744; doi:10.3389/fmicb.2016.02057)

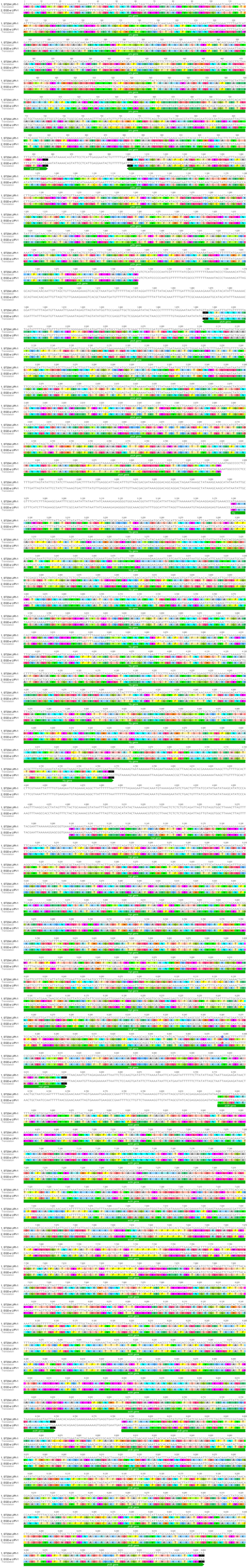

Supplement: Figure S1 — Nucleotide alignment of the conserved serotype 1/2a ST204 LIPI-1 pathogenicity island with the serotype 1/2a ST35 EGD-e reference strain. [file Image1.PDF]
